# Subinhibitory Concentration of Kanamycin Induces the *Pseudomonas aeruginosa* type VI Secretion System

**DOI:** 10.1371/journal.pone.0081132

**Published:** 2013-11-08

**Authors:** Cerith Jones, Luke Allsopp, Jack Horlick, Hemantha Kulasekara, Alain Filloux

**Affiliations:** 1 MRC-Centre for Molecular Bacteriology and Infection (CBMI), Department of Life Sciences, Imperial College London, London, United Kingdom; 2 Departments of Genome Sciences, Medicine, and Microbiology, University of Washington, Seattle, Washington, United States of America; Université d'Auvergne Clermont 1, France

## Abstract

*Pseudomonas aeruginosa* is a Gram-negative bacterium found in natural environments including plants, soils and warm moist surfaces. This organism is also in the top ten of nosocomial pathogens, and prevalent in cystic fibrosis (CF) lung infections. The ability of *P. aeruginosa* to colonize a wide variety of environments in a lasting manner is associated with the formation of a resistant biofilm and the capacity to efficiently outcompete other microorganisms. Here we demonstrate that sub-inhibitory concentration of kanamycin not only induces biofilm formation but also induces expression of the type VI secretion genes in the H1-T6SS cluster. The H1-T6SS is known for its role in toxin production and bacterial competition. We show that the antibiotic induction of the H1-T6SS only occurs when a functional Gac/Rsm pathway is present. These observations may contribute to understand how *P. aeruginosa* responds to antibiotic producing competitors. It also suggests that improper antibiotic therapy may enhance *P. aeruginosa* colonization, including in the airways of CF patients.

## Introduction

Bacteria have developed strategies to respond and adapt to variation in their surroundings. Probing the environment, transferring the information and implementing appropriate behaviour involve regulatory cascades that help bacteria in their decision making process [1]. The number and complexity of regulatory networks is proportional to the versatile potential of the organism. The *Pseudomonas aeruginosa* genome carries as much as 8% of genes involved in regulatory mechanisms [2,3]. *P. aeruginosa* is an ubiquitous bacterium found in several environments, e.g. soil, water, vegetation [4]. It is also a nosocomial pathogen affecting individuals defective in immune defences [5], and a major cause of morbidity and mortality, particularly with cystic fibrosis (CF) patients. Infection occurs at various places in the body, e.g. skin, eyes, lungs, further demonstrating the unique versatility and adaptive potential of this organism [6].

Like many other microorganisms, *P. aeruginosa* thrives as a motile organism, i.e. planktonic lifestyle, or establishes a bacterial community known as biofilm, i.e. sessile lifestyle. In biofilms the bacterial population is embedded in a matrix of exopolysaccharides and attached onto a surface [7]. The bacterial biofilm is resistant to stresses, including antibiotics or immune defences. This lifestyle is associated with persistent infection while planktonic lifestyle correlates with systemic dissemination [8]. The regulatory mechanisms, by which biofilm formation and dispersal are controlled, involve several regulatory cascades such as two-component systems, small regulatory RNAs and post-transcriptional regulators [9]. Furthermore, the switch between biofilm formation and dispersal is tightly linked to the intracellular concentration of the second messenger c-di-GMP [10]. Whereas the different components involved in these regulatory cascades are largely characterized, the specific mechanism by which these detect environmental stimuli such as nutrient limitation/availability, dehydration or redox condition is still poorly understood.

It has been observed that subinhibitory concentration of antibiotics can induce *P. aeruginosa* biofilm formation. This was shown for aminoglycosides [11], quinolones [12] and tetracycline [13]. However, this was not the case for all antibiotics, since polymixin B, chloramphenicol or carbenicillin did not have an impact on *P. aeruginosa* biofilm formation. This suggested that the response may be specific, and that these antibiotics may be used as signalling molecules rather than toxic compounds [13]. The main suggestion from the work by Hoffman and collaborators [11] was that aminoglycoside-dependent induction of biofilm formation was dependent on a gene, *arr*, for aminoglycoside response regulator, which encodes a phosphodiesterase [11]. Phosphodiesterases are involved in degradation of c-di-GMP. The observation that the Arr phosphodiesterase is needed for inducing biofilm is at odds with the general concept that high level of c-di-GMP promotes biofilm formation whereas phosphodiestereases reduce c-di-GMP concentration and induce biofilm dispersal [14,15]. We report here that the *arr* gene is not present in all *P. aeruginosa* strains, and for example is absent in the PAK strain.

One main regulatory network controlling the equilibrium between biofilm formation and dispersal is the Gac/Rsm pathway [16-21]. It was shown that this pathway not only impacts biofilm but is also an important regulatory switch for the antagonistic control of two protein secretion systems, the type III (T3SS) and the type VI secretion systems (T6SS). The T6SS was shown to be co-induced with determinants involved in biofilm formation in a Gac/Rsm-dependent manner, with concurrent down-regulation of the T3SS which is associated with virulence and cytotoxicity [22].

In the present study, we revisited the impact of subinhibitory concentration of antibiotics in the *P. aeruginosa* isolate PAK, which lacks the *arr* gene. We investigated more particularly the impact of the aminoglycoside kanamycin and extended the analysis to molecular determinants that are co-regulated with the biofilms such as the T6SS [23]. It is shown that at least one of the three T6SSs available to *P. aeruginosa*, the H1-T6SS, is associated with killing of bacterial competitors [24-27]. This mechanism may have an impact on modulating the composition of a polymicrobial environment or a mixed-species biofilm. Several reports suggest that bacteria in the environment may sense antibiotic producing organisms and mount an appropriate self-defence response against them [13]. The T6SS might be one of these defence mechanisms. Finally, we assessed whether the *P. aeruginosa* Gac/Rsm pathway, which co-regulates biofilm formation and T6SS expression, could be connected to the antibiotic-dependent response.

## Materials and Methods

### Bacterial strains and growth conditions

Bacterial strains used in this study are described in Table 1. *P. aeruginosa* strains were grown in LB or tryptone soy broth supplemented with antibiotics at 37 °C with agitation unless otherwise stated.

**Table 1:** Bacterial strains and Plasmids used in this study.

Strain or Plasmid	Characteristics	Source/Reference
*Pseudomonas aeruginosa*		
PAK	Wild-type *P. aeruginosa*	Laboratory collection
PAKΔH1-T6SS	In-frame deletion of H1-T6SS from mid *hsiA1* (PA0082) to mid *vgrG1a* (PA0091) in *Pseudomonas aeruginosa* PAK wild type strain	This study
PAKΔ*retS*	In-frame deletion of *retS* (PA4856) in *P. aeruginosa* PAK wild-type strain	[17]
PAKΔ*ladS*	In-frame deletion of *ladS* (PA3974) in PAK	[21]
PAKΔ*gacS*	In-frame deletion of *gacS* (PA0928) in PAK	[16]
PAKΔ*gacA*	In-frame deletion of *gacA* (PA2586) in PAK	[16]
PAKΔ*rsmYZ*	In-frame deletions of *rsmY* (PA0527.1) and *rsmZ* (PA3621.1) in PAK	[16]
PAK::pCTX-PA0082-*lacZ*	PAK carrying a PA0082-*lacZ* transcriptional fusion	[38]
PAKΔ*rsmA* ::pCTX-PA0082-lacZ	PAKΔ*rsmA* carrying a PA0082-*lacZ* Transcriptional fusion	[38]
PAK::pUC18-mini-Tn7-PA0082-*lacZ20*	PAK carrying a PA0082-*lacZ* translational fusion	[38]
PAK ΔrsmA ::pUC18-mini-Tn7-PA0082-*lacZ20*	PAKΔ*rsmA* carrying a PA0082-*lacZ* translational fusion	[38]
*Escherichia coli*		
One-shot® TOP10	Used for interbacterial competition assay. F- *mcr*A ∆(*mrr-hsd*RMS-*mcr*BC) φ80*lac*Z∆M15 ∆*lac*X74 recA1 *ara*D139 ∆(*araleu*)	Invitrogen
CC118 λpir	Host strain for pKNG101 replication; ∆(*ara-leu*) *araD* ∆*lacX74 galE galK-phoA20thi-1 rpsE rpoB argE* (Am) *recA1 Rfr*λ*pir*)	Laboratory collection
1047	*E. coli* carrying conjugative plasmid pRK2013	[64]
Plasmids		
pSB302	Based on pMP220 containing 211 bp region of *exoT* promoter-*lacZ* reporter.	[42]
pSB305	Based on pMP220 containing 219 bp region of *pcrD* promoter-*lacZ* reporter	[42]
p*cdrA::gfp* ^*S*^	pUCP22Not-PcdrA-RBS-CDS-RNaseIII-gfp(Mut3)-T0-1, Amp^R,^ Gm^R^	[28]
pKNG101 ΔH1-T6SS	pKNG101 containing mutator fragment for deletion of H1-T6SS cluster	[32]
pCR2.1	TA cloning vector, Ap^R^, Km^R^	Invitrogen

### Biofilm formation

Qualitative visualisation of biofilm formation was performed in glass test tubes. LB broth was inoculated to 0.1 OD_600_ of the relevant *P. aeruginosa* PAK strain, and 3 ml of culture, supplemented with antibiotic as described, was added to each test tube. Test tubes were incubated at 37 °C, static, for 16 hours. Following incubation, crystal violet stain (Merck) was added at 0.1 volumes per well and left for either for 10 minutes or 2.5 h at room temperature on a rocker. Liquid was removed by aspiration, test tube washed with water, and crystal violet staining was visualised and photographed.

Quantitative analysis of biofilm formation was performed in microtitre plates (Falcon). Wells were inoculated with bacterial culture at 0.1 OD_600_ in LB broth and supplemented with antibiotics as described. Plates were incubated wrapped in plastic wrap at 37 °C, static, for 16 hours. Following incubation crystal violet stain was added at 0.1 volumes per well and left for ten minutes at room temperature. Liquid was removed from each well by aspiration, wells were washed three times with water, and the remaining stained biofilm was dissolved in 95% ethanol for ten minutes with rocking. The optical density of the dissolved stain was measured by reading OD_600_.

### Measurement of c-di-GMP levels

Levels of c-di-GMP were measured using the c-di-GMP promoter *cdrA-gfp* fusion-encoding plasmid [28]. The plasmid was introduced into *P. aeruginosa* PAK by electroporation, and transformed cells were incubated overnight with gentamycin (100 µg/ml). Cells were subcultured to 0.1 OD_600_ the following day in the absence of antibiotics, or with kanamycin at 30 µg/ml for six hours at 37 °C with agitation. Following incubation cells were washed once in PBS, and 200 µl of cells were added to 96 well microtitre plates. Absorbance was measured at 620 nm, while fluorescence was measured by excitation at 485 nm and emission at 520 nm using a FLUOstar Optima plate reader. Fluorescence values were normalised to 1 ml and corrected for optical density.

### Immunoblots and analysis of protein from cell extracts

Cell extracts were prepared by harvesting 1 ml of bacterial culture in Tryptone Soy Broth by centrifugation. Cells were suspended to a density of 0.01 OD_600_ units per µl in Laemmli buffer, and boiled at 95 °C for ten minutes prior to separation by SDS-PAGE (loaded at 0.1 OD_600_ equivalent units per well). Proteins from cell extracts were transferred to nitrocellulose membrane.

Antibodies against the VgrG1a protein or a Hcp1 peptide were used at 1:1000 dilutions as previously described [29]. Anti PcrV antibody was used at 1:1000. Anti-RNA Polymerase antibody, directed against the *E. coli* beta subunit, was obtained from Neoclone and used at a dilution of 1:10,000. Primary antibodies were incubated for 1-2 hours at room temperature, followed by 45 minutes incubation with the appropriate secondary antibody (goat anti-rabbit HRP or rabbit anti-mouse HRP) at a dilution of 1:5000. Western blots were developed with SuperSignal West Pico chemiluminescent substrate (Pierce/Thermo) and quantified using Las3000 Fuji imager.

### Construction of *P. aeruginosa* deletion mutants

Deletion of DNA regions from the *P. aeruginosa* chromosome was performed as previously described [30] using the suicide vector pKNG101 [31]. Deletion of the H1-T6SS cluster in *P. aeruginosa* PAK was achieved by deletion of a region spanning from mid-PA0082 to mid-PA0091 (*hsiA1* to *vgrG1a*). This mutation was previously described in a PAKΔ*retS* background [32] and here we constructed the mutation in a PAK wild type background (Table 1). Plasmid pKNG-ΔH1-T6SS (Table 1) was maintained in *E. coli* CC118 λ*pir* and mobilised into *P. aeruginosa* PAK by three-partner conjugation using the *E. coli* 1047 helper strain. Double recombination events, resulting in the deletion of the H1-T6SS cluster were selected on sucrose plates and verified by PCR using external primers.

### PCR screening of *arr* genetic region

*P. aeruginosa* PAK genomic DNA was probed with primers specific to a 686 base pair fragment of the *arr* ORF from *P. aeruginosa* PAO1 strain; 5’-AGCGCATCACCCCCAGCAAC-3’ and 5’-CGCCAAGTGCGAGCCACTGA-3’, to investigate the presence or absence of this gene.

### Protein Secretion Assay

T3SS or T6SS secretion assays were performed under native, non-inducing conditions. Overnight cultures of *P. aeruginosa* strains grown in TSB broth were subcultured to OD_600_ of 0.1 and grown to early stationary phase (6 hours) at 37 °C with agitation. Cells were separated from culture supernatants by centrifugation at 4000 x *g* at 4°C. Proteins were precipitated using 6 M trichloroacetic acid at a final concentration of 10% (v/v). Following precipitation supernatant fractions were washed in 90% acetone, air dried, and resuspended in Laemmli buffer to a concentration of 0.1 OD_600_ equivalent units per µl, and loaded to 1.0 OD_600_ equivalent units for SDS-PAGE.

### β-galactosidase assay

*P. aeruginosa* strains carrying chromosomal *lacZ* fusions were grown overnight in LB and subcultured the following day to a density of 0.1 OD_600_ equivalent units in LB broth with kanamycin as indicated. Subcultures were incubated at 37 °C with agitation for 6 hours.

*P. aeruginosa* strains carrying T3SS *lacZ* fusion plasmids were grown overnight in LB broth supplemented with tetracycline (15 µg/ml) and subcultured the following day to 0.1 OD_600_ equivalent units in LB broth without tetracycline, but with kanamycin as indicated. Subcultures were incubated at 37 °C with agitation for 6 hours.

β-galactosidase activity was quantified as previously described [33].

### Bacterial competition assay

Bacterial competition assay was performed as previously described [32]. *P. aeruginosa* PAK was co-cultured with *E. coli* carrying the pCR2.1 vector, allowing alpha complementation for β-galactosidase. *P. aeruginosa* cells were incubated in a mixed patch with equivalent numbers of *E. coli* pCR2.1 cells on an LB plate, or LB plates supplemented with kanamycin at 50 µg/ml, for 5 hours at 37 °C. Following incubation the mixed patches of bacteria were recovered and resuspended in LB broth. A dilution series ranging from 10° to 10^-3^ was plated in triplicate onto LB plates supplemented with 100 µg/ml X-gal (Invitrogen). LacZ positive (blue) *E. coli* could then be detected after incubation at 37 °C overnight, giving a qualitative indication of *E. coli* survival.

## Results

### *Pseudomonas aeruginosa* PAK biofilm formation is induced by subinhibitory concentration of antibiotics

**Figure 1:**
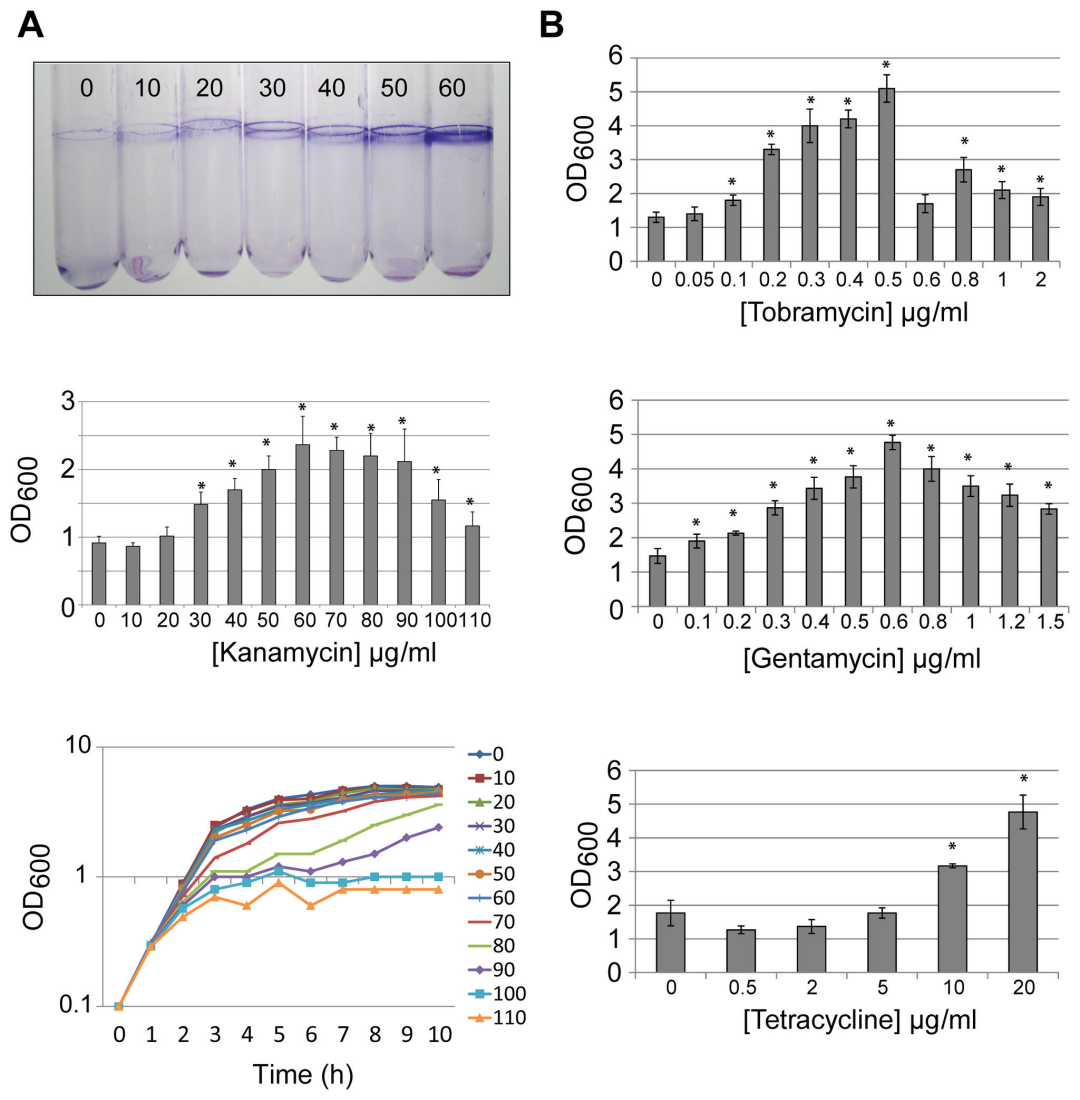
Induction of biofilm formation in *P. aeruginosa* by subinhibitory concentrations of antibiotics. (A) *P. aeruginosa* PAK biofilm formation upon addition of kanamycin. Top panel shows staining of biofilm biomass with crystal violet in glass test tubes. The concentration of kanamycin (in µg/ml) is indicated above each tube. Biofilm production is quantified by measuring the optical density (OD_600_) of the dissolved crystal violet (middle panel). Error bars show standard deviation. The kanamycin concentration used is indicated below each bar. * indicates significantly higher biofilm levels compared to PAK incubated without antibiotic (Student’s T Test, P<0.05). Planktonic growth (OD_600_) of PAK with increasing kanamycin concentration is shown over a ten hour period (lower panel). (B) Quantification of biofilm formation by PAK grown with increasing concentrations of tobramycin (top), gentamycin (middle) and tetracycline (lower). The concentration of each antibiotic used is indicated below each bar.

In a previous study it has been proposed that subinhibitory concentration of various antibiotics induces biofilm formation in *P. aeruginosa* PAO1. In particular, it was shown that tobramycin-induced biofilms were dependent on a phosphodiesterase, Arr [11]. It was proposed that in *P. aeruginosa* isolates not responding to tobramycin the *arr* gene was missing. Here we used our model bacterium, *P. aeruginosa* PAK, and showed that it is lacking the *arr* gene by PCR analysis on genomic DNA (data not shown). We tested a series of antibiotics, aminoglycosides or tetracycline, which were all shown previously to induce biofilm formation in other *P. aeruginosa* isolates [11]. Our data show that subinhibitory concentration of kanamycin consistently induces PAK biofilm as tested by the crystal violet method, with a maximum induction observed at a concentration of 60 μg/ml (Figure 1A). At this concentration the planktonic growth curve was only slightly affected, whereas at higher concentration of antibiotic a clear impact on growth could be observed (Figure 1A). The impact on biofilm formation was also observed with tobramycin and gentamycin, two other aminoglycosides, with a maximum peak at subinhibitory concentration of 0.5 and 0.6 μg/ml, respectively (Figure 1B). Finally, induction of biofilm formation could be observed by using a non-aminoglycoside antibiotic, such as tetracycline (Figure 1B). In conclusion, we observed that *P. aeruginosa* PAK responds to subinhibitory concentration of antibiotic and forms biofilm independently of the *arr* gene.

### Kanamycin-dependent induction of biofilm formation does not correlate with a detectable change in c-di-GMP level

**Figure 2:**
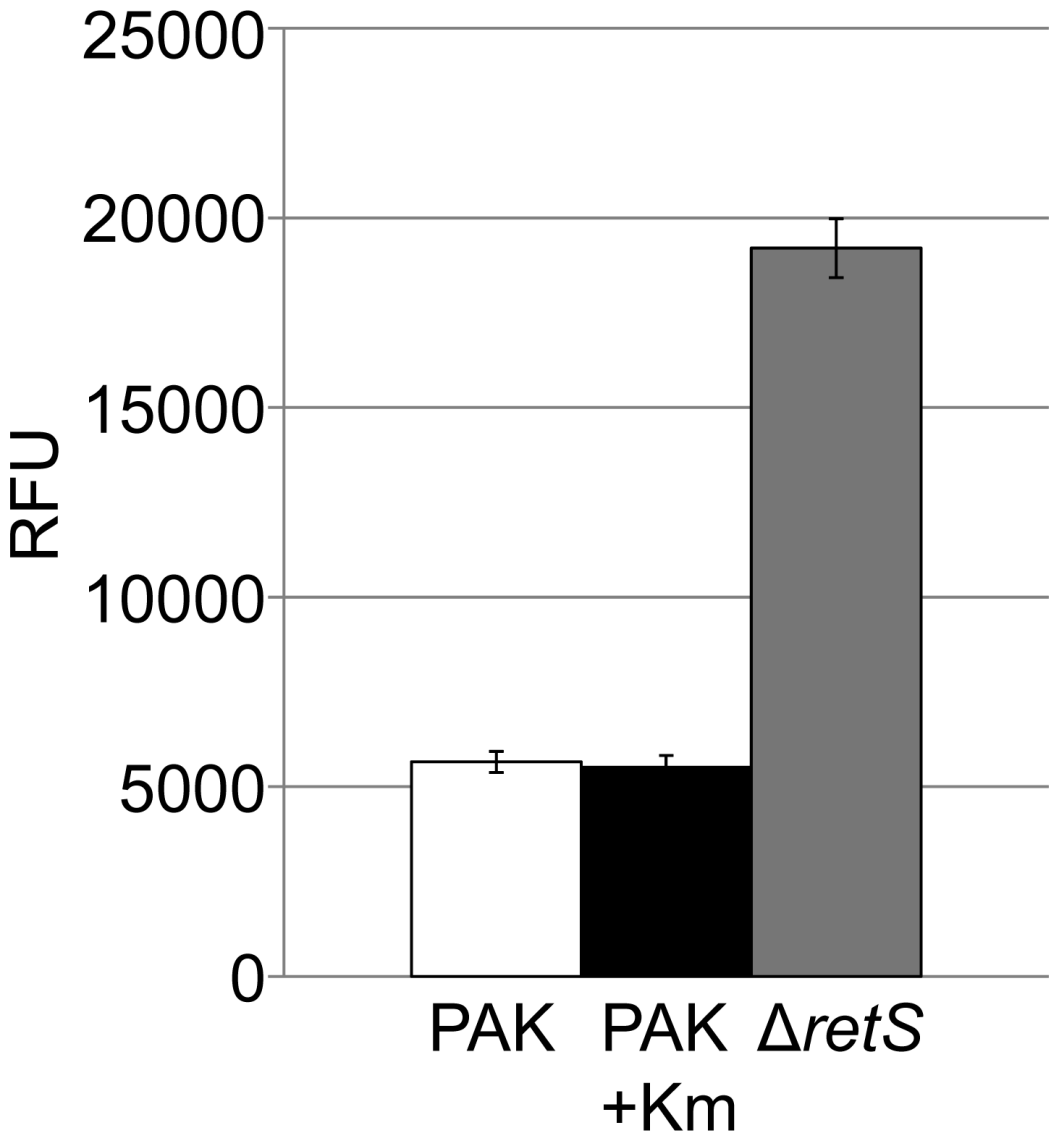
Analysis of c-di-GMP levels in *P. aeruginosa* PAK strains. Relative fluorescence values are shown for strains carrying the c-di-GMP reporter plasmid (*cdrA-gfp* promoter fusion) [28]. Levels are shown for wild type PAK, PAK incubated with kanamycin at 30 µg/ml, and a control strain (PAKΔ*retS*) known to have elevated c-di-GMP levels [35]. RFU indicates arbitrary relative fluorescence units corrected for optical density. Error bars show standard deviation.

It is generally accepted that formation of biofilm in Gram-negative bacterium, such as *P. aeruginosa*, correlates with an increase in c-di-GMP level [34]. However, the studies highlighting the role of Arr in the antibiotic-dependent induction of biofilm suggested that in this case the induction correlates with a decrease in c-di-GMP since Arr is a phosphodiesterase [11]. In the present study, we used a transcriptional fusion, *cdrA-gfp* [28], which responds to increase in c-di-GMP levels. We observed that addition of kanamycin does not induce any changes in the level of Gfp-related fluorescence (Figure 2), suggesting that kanamycin-dependent biofilm induction is not connected with the c-di-GMP second messenger or that the change in c-di-GMP level sufficient to induce a switch in biofilm behaviour are below the level of detection using this method. As a control we have used a strain in which the *retS* gene is deleted, which results in c-di-GMP increase and hyperbiofilm formation (Figure 2) [35].

### Sub-inhibitory kanamycin concentration induces H1-T6SS

**Figure 3:**
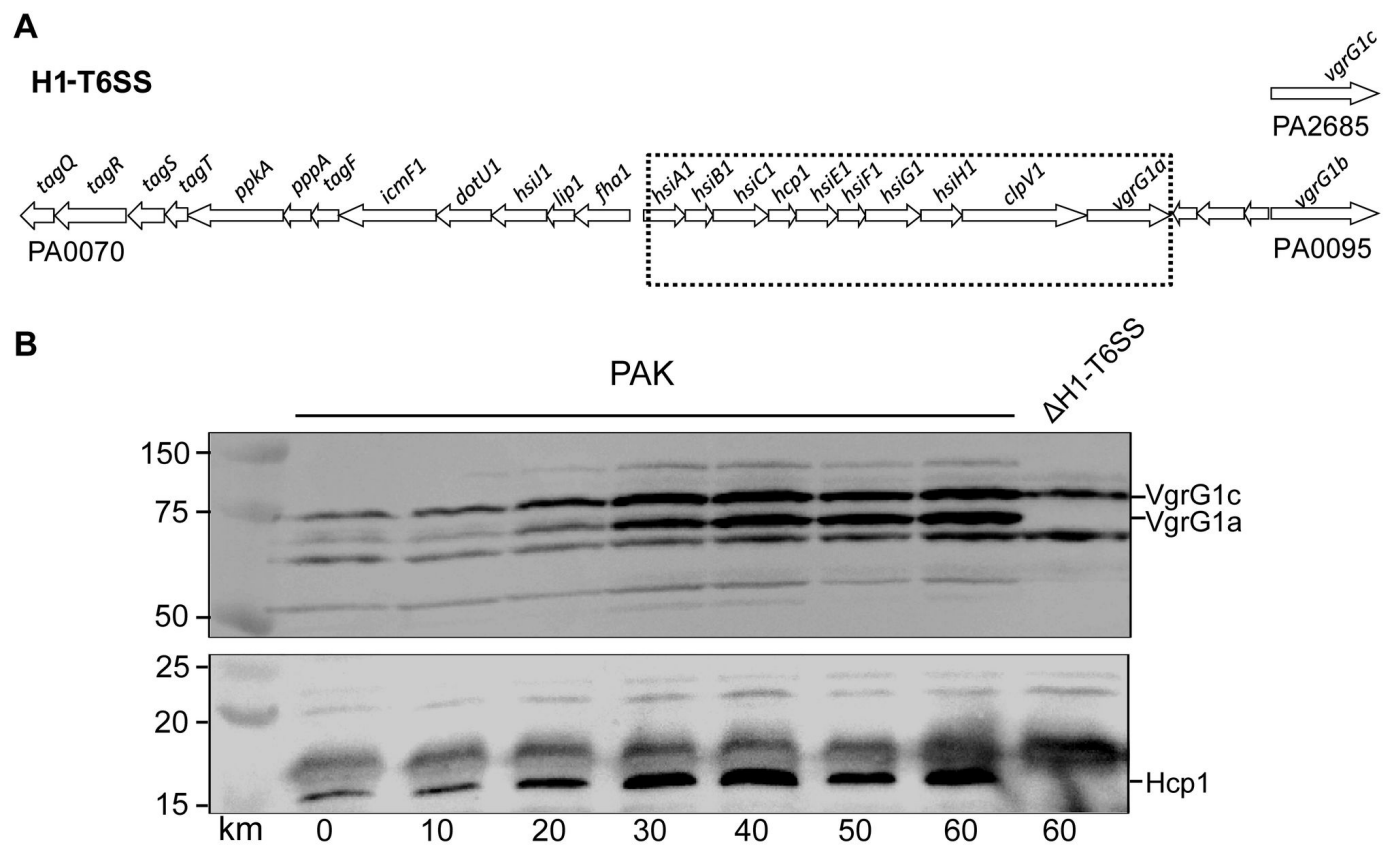
Kanamycin induces components of the H1-T6SS in *P. aeruginosa* PAK. (A) Genetic organisation of the H1-T6SS cluster. The PAKΔH1-T6SS mutant has a deletion encompassing the *hsiA1* to *vgrG1a* genes as indicated by the hatched box. (B) Western blot of PAK whole cell extracts from cells incubated with increasing concentrations of kanamycin, indicated in µg/ml below the blots. Cell extracts were probed with anti-VgrG1a (upper blot) and Hcp1 (lower blot). The expected position of the proteins of interest is indicated on the right of the blot, and molecular weight markers (in kDa) are indicated on the left. Cell extract from the PAKΔH1-T6SS mutant is included as a control to positively identify VgrG1a and Hcp1, which are encoded within the H1-T6SS cluster and thus absent in this strain (right hand lane).

It is well documented that biofilm formation in *P. aeruginosa* is co-regulated with H1-T6SS activity [35,36]. We assessed whether H1-T6SS is also induced by subinhibitory concentration of antibiotics. We grew the PAK strain in media containing various concentration of kanamycin and tested the induction of two known components coded by the H1-T6SS cluster (Figure 3A), Hcp1 and VgrG1a [29]. Immunoblots on proteins from whole cell extracts showed that bands could be detected with increased intensity for both components, and the gradual increase in the bands intensity reaches a maximum at a concentration of 60 μg/ml (Figure 3B), similar to what was observed with the biofilm phenotype. It should be noted that the antibody against VgrG1a recognizes both VgrG1a and VgrG1c (Figure 3B) as previously reported [29]. The detection of VgrG1a and Hcp1 is specific since it is only observed in the PAK strain and not in a mutant in which most of the H1-T6SS gene cluster has been deleted (Figure 3A and 3B). We concluded that co-regulation of biofilm and T6SS induction, which has been reported before [35], is also observed when *P. aeruginosa* is exposed to subinhibitory concentration of antibiotics.

We also tested the impact of subinhibitory kanamycin concentration on activation of H1-T6SS using the PAO1 strain. Intriguingly, we observed that this strain has a higher background in terms of basal level of Hcp1 production, which prevented the observation of a quantifiable impact upon addition of kanamycin (data not shown).

### A functional LadS/Gac/Rsm pathway is needed for the antibiotic dependent response

**Figure 4:**
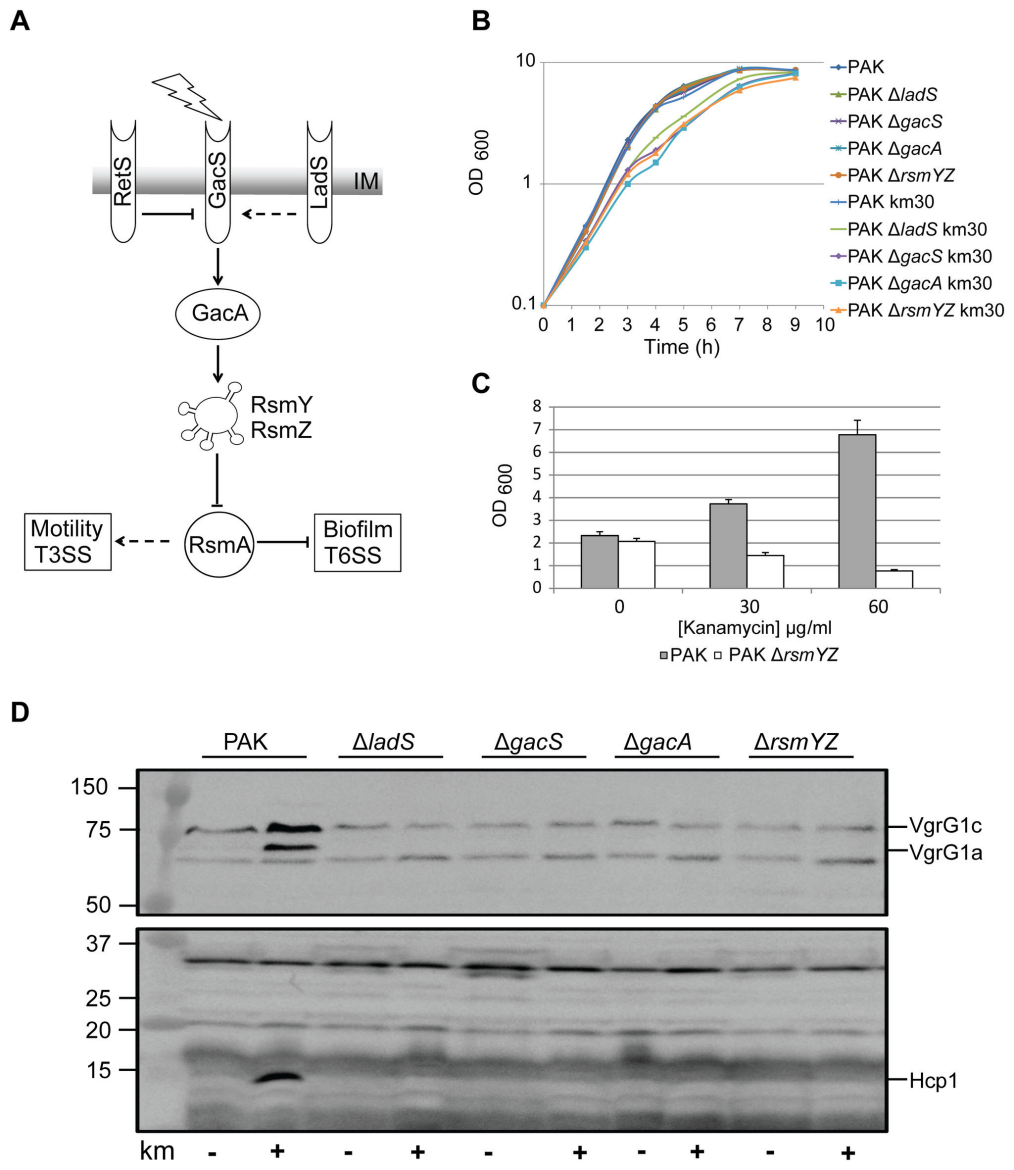
Kanamycin induction of H1-T6SS requires a functional LadS/Gac/Rsm pathway. (A) Schematic representation of the Gac/Rsm signalling cascade which antagonistically controls the phenotypic switches biofilm/motility and T6SS/T3SS. RetS, GacS and LadS are inner membrane located sensors, GacA is a cytoplasmic response regulator, RsmYZ are small RNAs and RsmA a translational repressor. Pointed arrows indicate a positive effect, while blocked arrows indicate a negative effect. Dashed lines indicate an indirect interaction. An unknown activating signal is represented by the lightning bolt. (B) Planktonic growth of PAK and isogenic mutants in components of the LadS/Gac/Rsm cascade with and without kanamycin at a concentration of 30 µg/ml. The black arrow indicates sampling time for cells and supernatants used in Figure 4D. (C) Biofilm formation upon addition of kanamycin to PAK and PAK Δ*rsmYZ*, as indicate by the key below the graph. (D) Western blot of whole cell extracts from PAK and isogenic deletion mutants lacking genes encoding components of the regulatory pathway as indicated above the blot. Strains were incubated with or without kanamycin at 30 µg/ml as indicated below the blot. Cell extracts were probed with anti-VgrG1a (upper blot) and Hcp1 (lower blot). The expected positions of the proteins of interest are indicated on the right of the blot, and molecular weight markers (in kDa) are shown on the left.

The Gac/Rsm pathway is a central regulatory cascade that controls biofilm formation and H1-T6SS activity [35] (Figure 4A), and which is positively influenced by the LadS hybrid sensor [21]. The Gac two-component system induces the expression of two small RNAs, RsmY and RsmZ, which in turn sequester the post-transcriptional repressor RsmA [19,37]. It was shown that RsmA binds to *H1-T6SS* mRNA and prevents its translation [38]. Here we further checked whether the kanamycin induction of biofilm or of the H1-T6SS depends on the Gac/Rsm pathway. We observed that a functional signalling cascade is required since a mutant in the small RNA genes *rsmY* and *rsmZ* do not form biofilm even upon addition of kanamycin (Figure 4C). Furthermore we observed that either *gacS*, *gacA* or *rsmY*/*rsmZ* mutants do not respond to kanamycin induction as seen by the absence of Hcp1 or VgrG1a in the cell extracts under these growth conditions (Figure 4D), whereas growth of all strains is not significantly impacted by addition of kanamycin at 30 μg/ml (Figure 4B).

**Figure 5:**
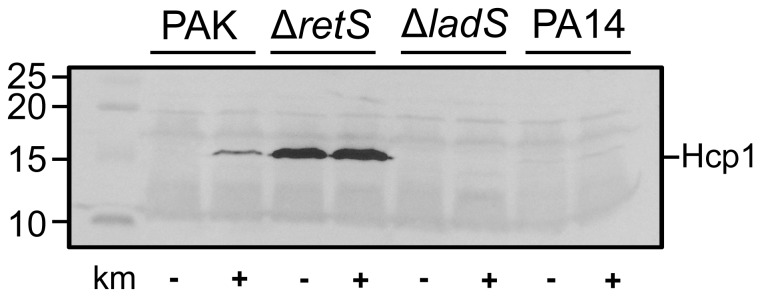
Impact of kanamycin on Hcp1 production in various *P. aeruginosa* backgrounds. Western blot showing Hcp1 production in whole cell lysates of *P. aeruginosa* PAK, PAKΔ*retS*, PAKΔ*ladS* and *P. aeruginosa* PA14. Each strain was grown with or without kanamycin at 30 µg/ml as indicated below the blots (+/-). The position of Hcp1 is indicated on the right of the blot, and molecular weight markers (in kDa) indicated on the left.

**Figure 6:**
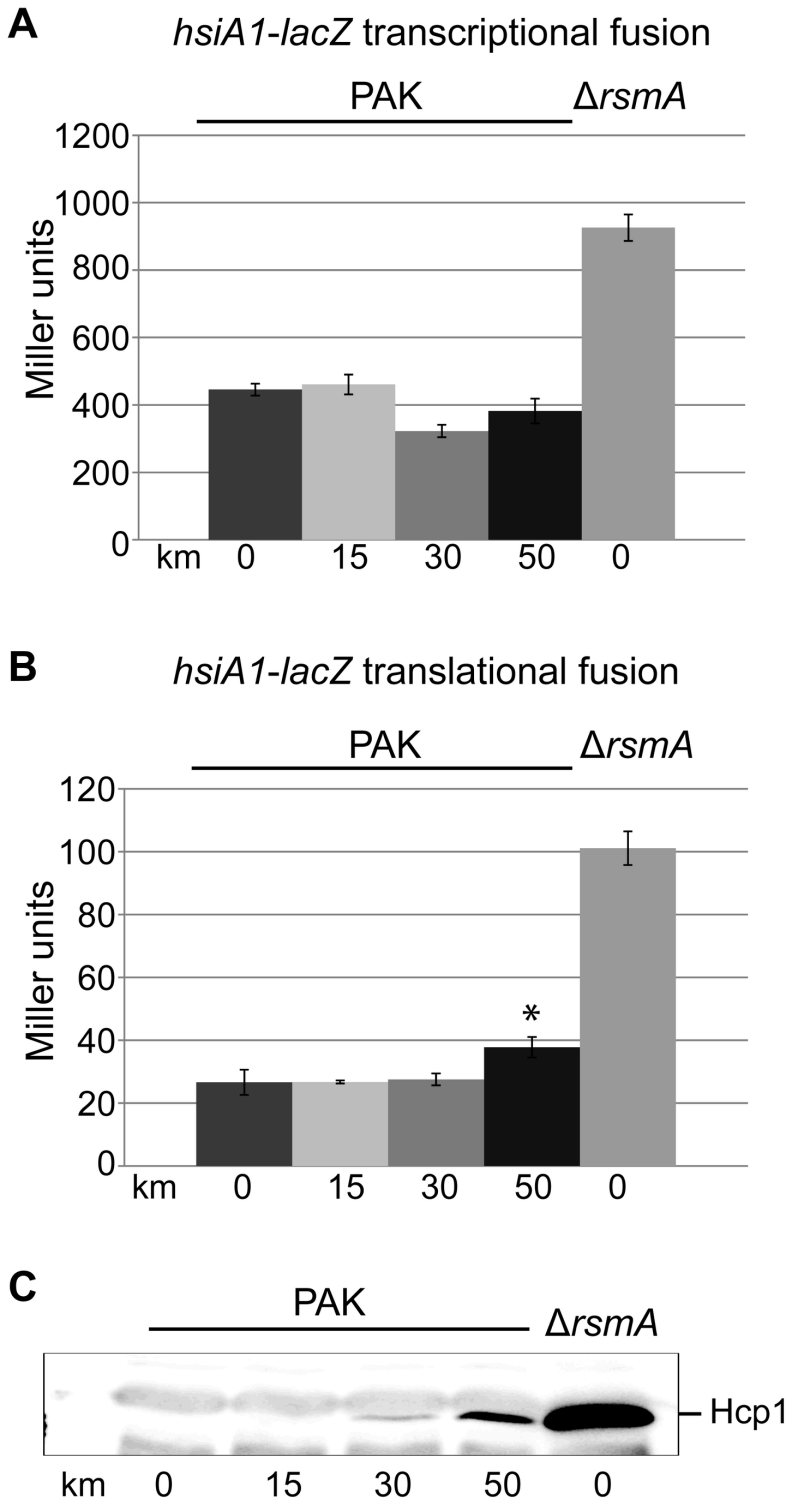
Effect of kanamycin on expression of PA0082 (*hsiA1*). Levels of β-galactosidase activity in PAK carrying chromosomal transcriptional (A) and translational (B) fusions of the *hsiA1* gene (PA0082) to *lacZ* [38] with increasing concentrations of kanamycin as indicated in µg/ml on the x axis. Both fusions introduced in a PAKΔ*rsmA* mutant were included as a positive control showing increased transcription and translation in this background as shown previously [38]. The * indicates a reproducible statistically significant difference (Student’s T test, P<0.05) compared to incubation without kanamycin. (C) Western blot showing induction of Hcp1 production in the translational fusion strain under corresponding kanamycin concentrations.

Interestingly, even a mutation in the *ladS* gene, which is upstream of the GacS sensor, resulted in the absence of production of H1-T6SS components in response to kanamycin (Figure 4D). This is in good agreement with our previous report about the *P. aeruginosa* PA14 strain, which is a spontaneous *ladS* mutant [39]. Here we further show that addition of sunbinhibitory concentration of kanamycin to a PA14 culture did not result in increase of H1-T6SS as seen in western blot using antibodies against Hcp1 (Figure 5). This is in contrast to what is observed with PAK and similar to the PAKΔ*ladS* mutant (Figure 5). In a PAKΔ*retS* mutant, Hcp1 production is already high and no further increase can be seen upon addition of kanamycin (Figure 5). We concluded that the LadS/Gac/Rsm signalling pathway needs to be fully functional in order to observe the response to subinhibitory concentration of kanamycin. We further analysed *hsiA1-lacZ* transcriptional or translational fusion [38] with *hsiA1* being the first gene of the H1-T6SS operon [40]. No dramatic increase in expression was detectable upon addition of kanamycin, except for a slight but significant impact on the translational fusion at 50 μg/ml kanamycin (T-test P<0.05 for two independent experiments) (Figure 6).

One observation we made is that the *gac* or *rsm* mutants appeared to be slightly more sensitive to kanamycin as compared to their parental strain (data not shown). Similar observation was reported previously with a *gacS* mutant, which has a MIC for gentamycin 4-fold lower as compared to PAK [41]. In this previous study, lower MICs were also observable for amikacin, chloramphenicol or cefpirome. A general stress imposed by antibiotic treatment and a slight effect on growth may have been responsible for the bacterial response rather than the antibiotic itself being the signalling molecule. However, the fact that in our hands kanamycin and tetracycline do similarly impact the growth (data not shown), but only kanamycin, and not tetracycline (data not shown), influences the T6SS response, is not in favor of this hypothesis. Furthermore we observed that at a concentration of kanamycin, which resulted in similar growth for both the PAK strain and isogenic *gac*/*rsm* mutants, the T6SS response is only observed in the parental strain (Figure 4).

### Kanamycin can induce the T6SS/T3SS switch

**Figure 7:**
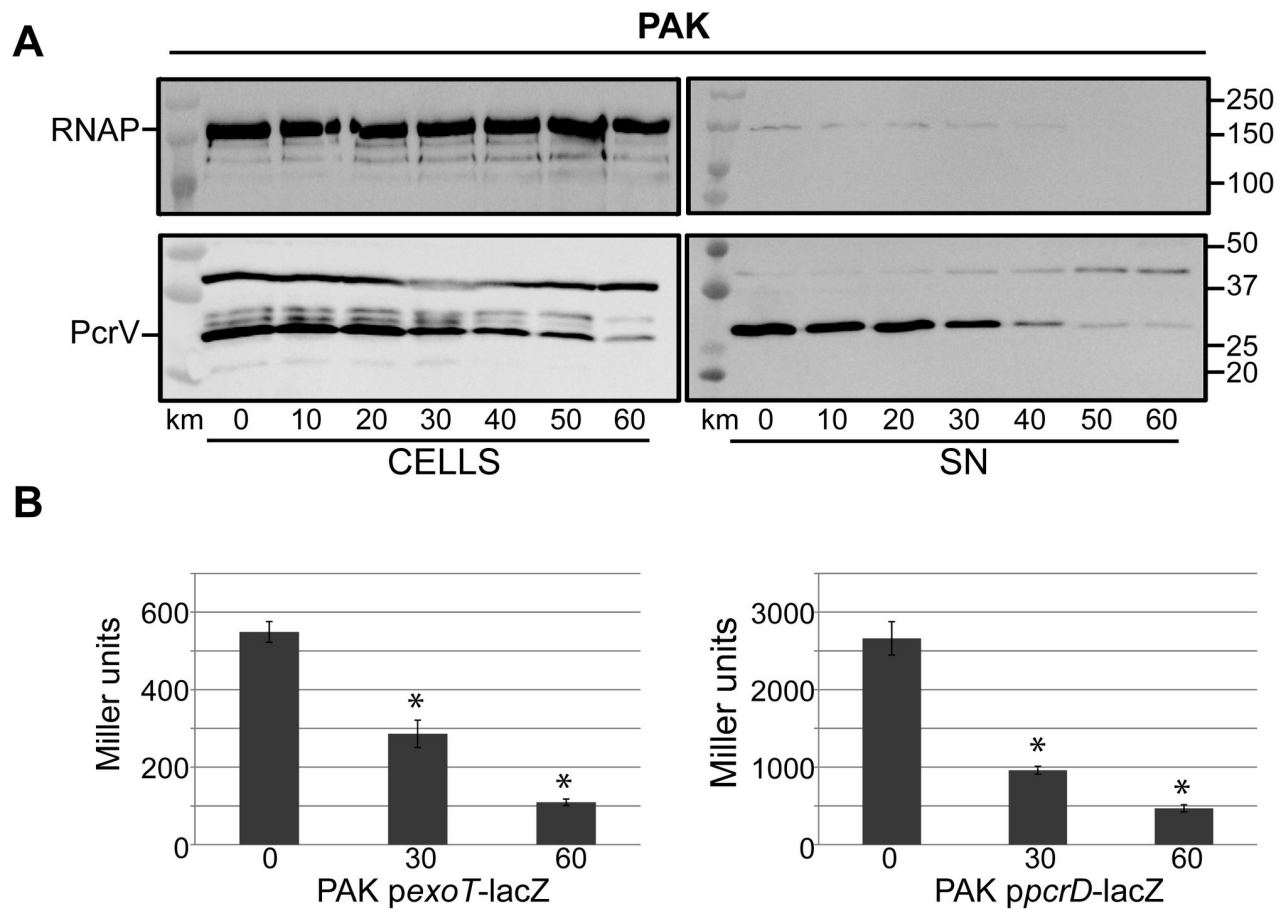
Kanamycin represses the T3SS. (A) Western blot analysis of whole cell extracts (left) and supernatant (SN) fractions (right) from *P. aeruginosa* PAK cells grown under increasing levels of kanamycin (µg/ml) as indicated below the blots. Upper blots are probed with anti-RNA polymlerase (RNAP), and lower blots probed with anti-PcrV. The expected position of these components is indicated on the left side of the blot, while molecular weight markers (in kDa) are shown on the right. (B) Effect of kanamycin on the activity of *lacZ* transcriptional fusions with *exoT* or *pcrD* promoter. β-galactosidase activity (Miller units) is shown for PAK strains carrying plasmid borne transcriptional fusions, incubated with increasing kanamycin concentration (µg/ml) indicated on the x-axis. * indicates a statistically significant difference in activity compared to the strain incubated without kanamycin. Error bars show standard deviation.

It is also known that the Gac/Rsm pathway antagonistically controls two secretion systems, i.e. the type III secretion system (T3SS) and the T6SS [17,35]. We performed immunoblot analysis on cell extracts and culture supernatants using antibodies directed against the T3SS component PcrV, which is at the tip of the T3SS needle. In agreement with the previously reported antagonism between T3SS and T6SS, we showed that increase in kanamycin concentration correlated with a gradual decrease in the amount of PcrV found either in the cell or the supernatant (Figure 7A). We also analysed the activity of T3SS*-lacZ* transcriptional fusions and confirmed that addition of kanamycin resulted in about 5-fold reduction in the level of β-galactosidase activity when using two such fusions, i.e. *exoT-lacZ* and *pcrD-lacZ* (Figure 7B) [42]. We concluded that kanamycin induces the H1-T6SS whereas it down-regulates the T3SS, similar to that observed by Gac/Rsm-dependent regulation.

### Does kanamycin-induced H1-T6SS promote bacterial killing?

**Figure 8:**
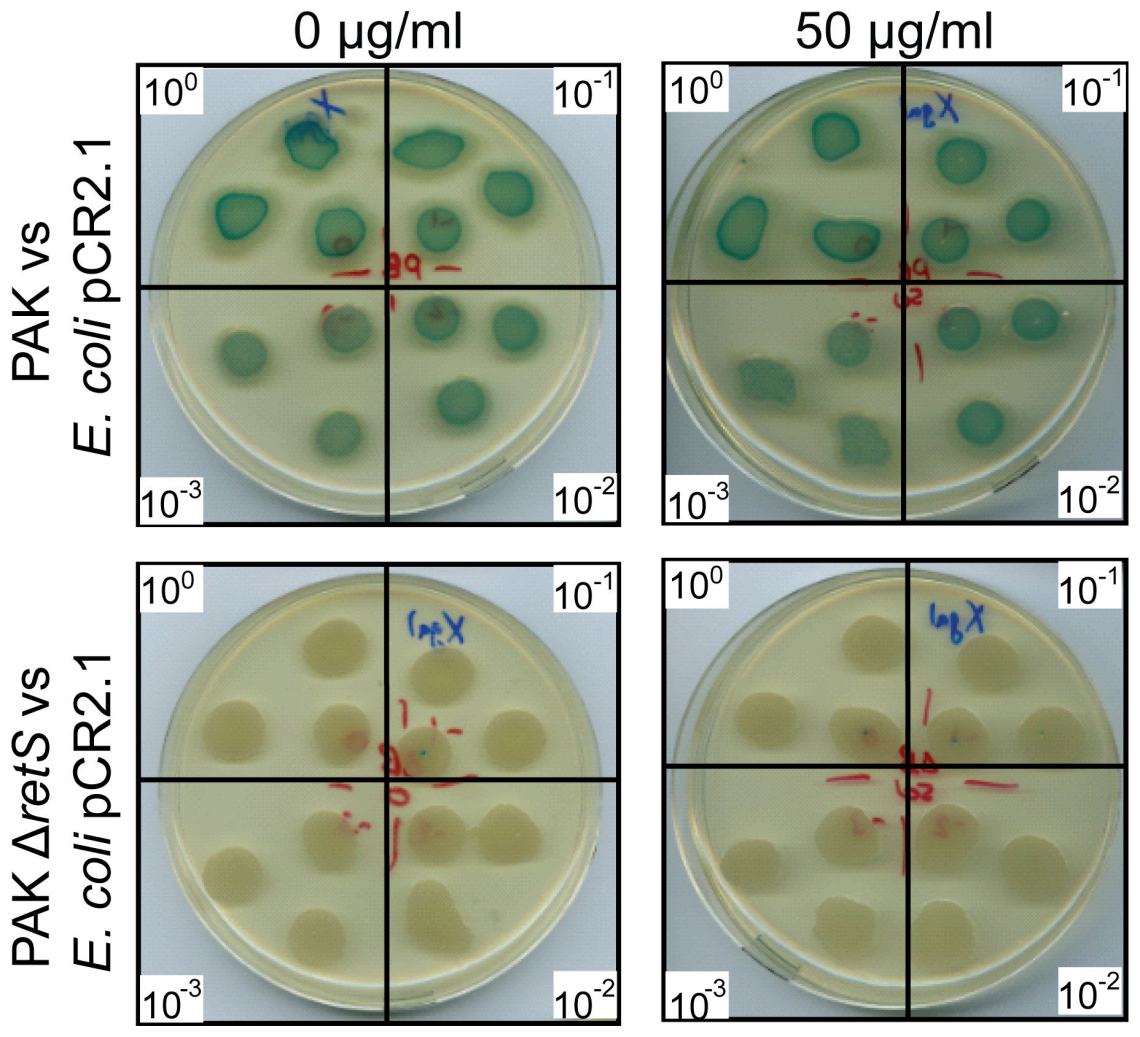
Kanamycin does not improve *P. aeruginosa* H1-T6SS mediated bacterial competition. *E. coli* cells carrying the pCR2.1 vector were co-incubated with either *P. aeruginosa* PAK wild type (top row), or PAKΔ*retS* (bottom row). The competition assay was carried out as previously described [32] on LB plates without antibiotics, or in the presence of kanamycin at a concentration of 50 µg/ml for five hours. Following competition cells were harvested and resuspended in LB broth, and a dilution series ranging from 10° to 10^-3^ was plated in triplicate onto LB plates, as indicated in the white boxes in each corner. Competition assays performed without kanamycin are shown on the left, while those with kanamycin are on the right. Visualisation of a blue colour gives a qualitative indication of *E. coli* survival.

One main function of the H1-T6SS is to secrete bacterial toxins and kill potential bacterial competitors [26]. We developed a visual assay to monitor T6SS-dependent killing of *Escherichia coli* by *P. aeruginosa* [32]. This is based on mixed culture of these two bacteria spotted on plates and monitoring the stability of *E. coli* in the mixed population by using X-gal as an indicator. *E. coli* produces the β-galactosidase and thus a mixed colony containing *E. coli* produces a dominant blue colour. Here we assessed whether addition of subinhibitory concentration of kanamycin in the plates increases *E. coli* killing by *P. aeruginosa*. However, no difference was observed at any of the kanamycin concentrations tested and *E. coli* survival was similar to what was observed in the absence of kanamycin (Figure 8).

**Figure 9:**
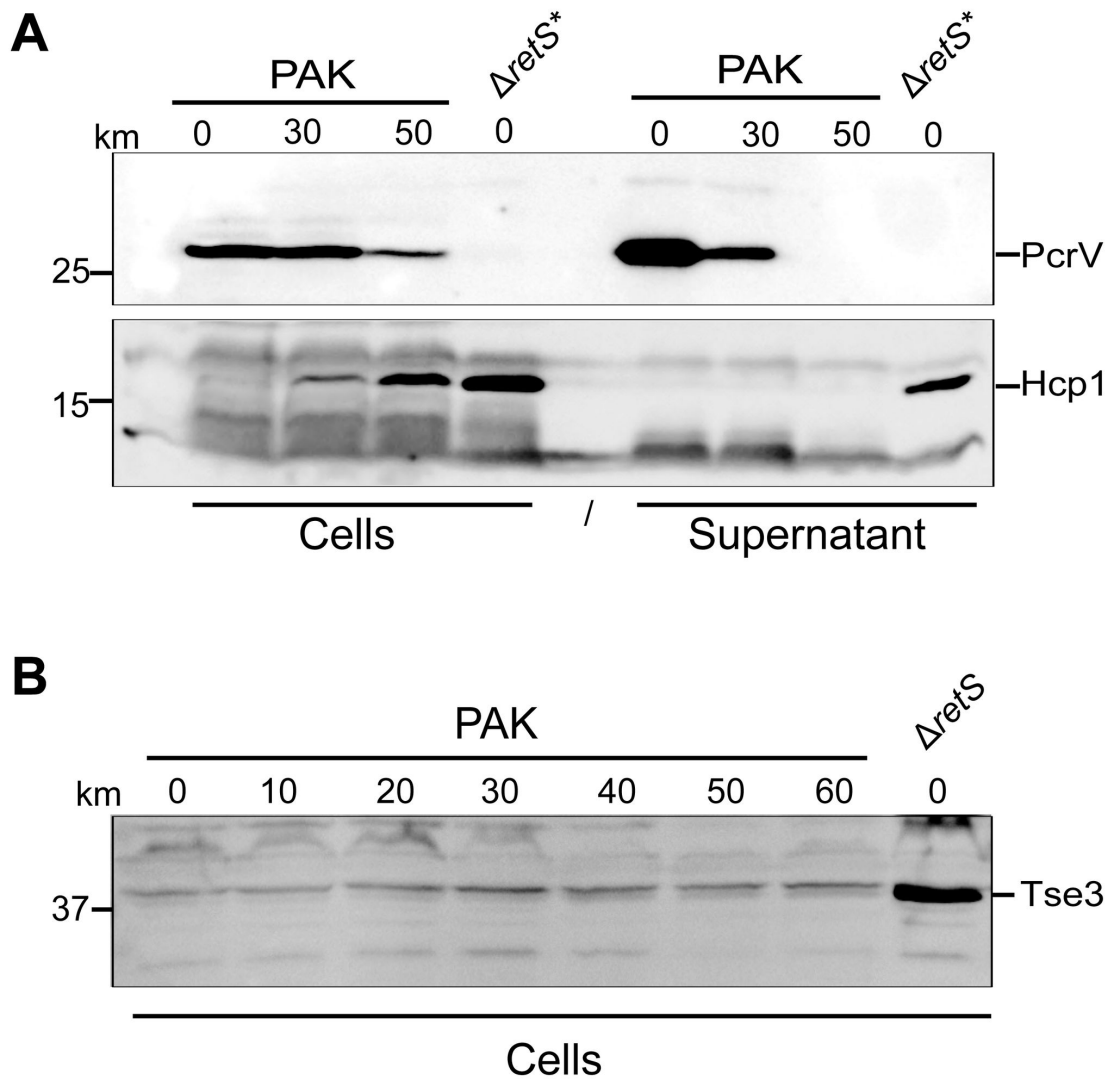
Kanamycin does not induce H1-T6SS-dependent secretion. (A) Secretion assay performed on PAK cells incubated with increasing concentrations of kanamycin as indicated above the blot in µg/ml. Whole cell extracts are shown on the left of the blot, while supernatants are shown on the right. Blots have been probed with anti-PcrV (upper blot) and anti Hcp1 (lower blot). The expected location of each protein is indicated on the left. The PAKΔ*retS* mutant strain, with a constitutively active H1-T6SS, is included as a positive control for secretion. * denotes that these samples have been diluted 1 in 10 with loading buffer to reduce the Hcp1 signal. (B) Western blot performed with anti-Tse3 antibodies on whole cell extracts prepared from PAK cells grown with increasing concentrations of kanamycin as indicated above the blots in µg/ml. PAKΔ*retS* is included as control as it is known that Tse3 is induced in this background.

We further checked whether addition of kanamycin induces H1-T6SS-dependent secretion. We performed immunoblots using Hcp and VgrG1a specific antibodies as described before and tested the occurrence of the proteins not only in the cell but also in the culture supernatant. In contrast to the T3SS component PcrV, we observed no Hcp1 secretion (Figure 9A), which suggests that the T6SS machinery may be assembled but not active.

The T6SS-dependent killing activity of *P. aeruginosa* is mostly dependent on the production of three bacterial toxins, Tse1, Tse2 and Tse3 [24]. We also tested the occurrence of Tse3 using a specific antibody and did not detect production of the toxin (Figure 9B). We thus concluded that kanamycin addition induces production of some T6SS components but is not sufficient to promote the formation of a fully active H1-T6SS.

## Discussion

*P. aeruginosa* is a bacterium that thrives in multiple environmental niches including soil, moist surfaces or plants but is also an opportunistic human pathogen [8]. Epidemiological studies showed no difference in the virulence potential of clinical or environmental isolates suggesting that the arsenal of virulence factors, including the T3SS and T6SS, are useful to fight eukaryotic predators in the environment (e.g. protozoans) [43]. *P. aeruginosa* is a versatile organism and its adaptive potential to environmental changes relies on signalling pathways, which determine whether *P. aeruginosa* adopts a planktonic/virulent or biofilm lifestyle [1,9]. The decision making process is governed by antagonistic pathways, which intersect via positive and negative feedback loops. Quorum Sensing and c-AMP signalling mostly promote virulence [44-46], whereas Gac/Rsm and c-di-GMP signalling pathways influence biofilm formation [10,17,47-49].

In this study we built up on previous observations showing that antibiotics induce biofilm formation and thus serve as signalling molecules [11,13]. It is unclear whether the response is specific to a particular category of antibiotics. Aminoglycosides but also non-aminoglycosides, such as tetracycline, induce biofilm formation [13]. In contrast polymixin B, carbenicillin or chloramphenicol have no effect on biofilm. Interestingly, antibiotics of the quinolone family, such as nalidixic acid, induce biofilm formation at concentration of 30 μg/ml [12], and this induction seemed to be correlated with an inhibition in DNA replication. It is a possibility that in this case the antibiotic molecule is not directly sensed by the bacterium but instead default replication induces the SOS response, which in turn activates regulatory pathways responsible for the increase level in biofilm formation.

Biofilm induction upon aminoglycoside [11] or nalidixic acid addition [12] appeared to rely on the phosphodiesterase Arr, thus suggesting that this phosphodiesterase is central to the antibiotic response. These previous experiments were performed using PAO1 as model organism but the *arr* gene is absent in the *P. aeruginosa* PA14 genome [50]. Among the 11 sequenced *P. aeruginosa* isolates (www.pseudomonas.com), *arr* is found in only two other cases, i.e. *P. aeruginosa* M18 and PA7 [51]. We showed by PCR amplification from genomic DNA that the widely used PAK isolate is also lacking the *arr* gene.

Our data challenge previous observations since we show that in PAK, a strain lacking *arr*, biofilm induction in response to a wide range of antibiotics, including aminoglycosides and tetracycline, is observed. We tested whether the response was associated with changes in c-di-GMP levels but did not see significant variation in concentration of this secondary messenger in response to antibiotics. Instead, what we observe is that the T6SS response to kanamycin requires a functional Gac/Rsm cascade including the LadS sensor, which is at the top of this regulatory network known to control biofilm formation. The two sensors, LadS and RetS, act upstream of Gac/Rsm and antagonistically control the activity of GacS. Whereas RetS negatively influences GacS [18], LadS has a positive effect and promotes biofilm formation [21]. It remains a possibility that LadS senses molecules such as aminoglycosides, since both RetS and LadS have sensing domains that are similar to carbohydrate binding modules (CBM) [52,53]. However, the relatively non-specific response to a wide range of antibiotics is not in favour of this hypothesis. Alternatively, one possibility is that the repression exerted by RsmA in mutants affected for the Gac/Rsm cascade cannot be alleviated and results in a dominant effect. In these conditions, the T6SS response upon addition of antibiotics cannot be observed in *gac/rsm* mutants due to direct repression by RsmA.

The Gac/Rsm pathway also influences expression of genes that are not required for biofilm, i.e. T6SS and T3SS [17,35]. Remarkably, we show that kanamycin positively influences the *P. aeruginosa* H1-T6SS, as seen by the induction of T6SS components such as Hcp1 and VgrG1a. We further confirm that the kanamycin induction mimics an activation of the Gac/Rsm pathway by demonstrating that addition of this antibiotic not only increases T6SS but simultaneously decreases T3SS, as does the Gac/Rsm cascade.

In contrast to biofilm formation, tetracycline, or aminoglycosides, such as tobramycin and gentamycin, did not significantly influence T6SS expression suggesting that the antibiotic response involves different regulatory circuits, such as the SOS response in case of nalidixic acid [12] or other antibiotics [54]. Impact of aminoglycosides on oxidative stress or iron uptake has been previously proposed, suggesting additional levels of complexity in the regulatory networks [55]. Furthermore, the PAO1 transcriptional profile assessed with a mini-array of 555 genes also showed variability and for each antibiotic tested a distinct profile was obtained [13]. The data presented in this publication are difficult to reconcile with our observation describing the role of Gac/Rsm in antibiotic response, since for example the *rsmA* gene is up-regulated (3.12-fold) in presence of tobramycin while it is down regulated (1.42-fold) in presence of tetracycline, but in both cases biofilm is induced and only in the case of tetracycline T3SS is increased [13]. In theory, it is expected and reported that an *rsmA* mutant has a hyperbiofilm phenotype [56,57] and a likely explanation is the involvement of multiple pathways depending on the antibiotic and the strain used.

The phenotypes we see might result from a direct response of *P. aeruginosa* when encountering antibiotics as signalling molecules. We can hypothesize that *P. aeruginosa*, originally a soil bacterium, frequently shares its ecological niche with Streptomycetes species, which are heavy antibiotic producers [58]. As part of the arsenal available to fight against other bacteria, *P. aeruginosa* can use pyocyanin [59] but also the T6SS, which is involved in the production of bacterial toxins named Tse1-3 [24], although it is unclear whether T6SS has any impact on Gram-positive bacteria. We observed Streptomyces killing by *P. aeruginosa*, but this was indeed independent of a functional H1-T6SS (data not shown). Nevertheless, we also tested the influence of kanamycin on *P. aeruginosa* killing activity using *E. coli* as a target cell. We were not able to see any kanamycin-dependent killing in these conditions. Intriguingly, we observed that kanamycin addition, despite inducing Hcp and VgrG1a, did not efficiently result in production of Tse3, which might explain the absence of killing. The T6SS machinery can thus be expressed upon antibiotic addition but the secretion might not be active. It has been shown that a regulatory network involving the PpkA kinase exerts a tight post-translational control on the T6SS activity [60-62], and thus despite inducing production of T6SS components, kanamycin addition may have no impact on PpkA activation, which will require another signalling mechanism such as cell-cell contact [63]. In other words, having sensed the presence of competing organisms and producers of antibiotics such as kanamycin, PAK is primed and ready to initiate an H1-T6SS-dependent attack.

In conclusion we have confirmed that antibiotics can act as signalling molecules and induce novel phenotypes in *P. aeruginosa* suggesting bacteria can detect antibiotic producers in the environment. These new phenotypes, such as biofilm and T6SS, can provide *P. aeruginosa* a clear growth advantage and resistance to additional stress. We showed that a specific response could be observed when using kanamycin. Kanamycin-dependent response includes induction of biofilm and T6SS, and repression of T3SS. This response relies on a functional Gac/Rsm pathway.
